# Associations of firearm dealer openings with firearm self-harm deaths and injuries: A differences-in-differences analysis

**DOI:** 10.1371/journal.pone.0248130

**Published:** 2021-03-18

**Authors:** Ellicott C. Matthay, Kriszta Farkas, Dana E. Goin, Kara E. Rudolph, Veronica A. Pear, Jennifer Ahern

**Affiliations:** 1 Center for Health and Community, School of Medicine, University of California, San Francisco, San Francisco, California, United States of America; 2 Division of Epidemiology & Biostatistics, School of Public Health, University of California, Berkeley, Berkeley, California, United States of America; 3 Program on Reproductive Health and the Environment, Department of Obstetrics, Gynecology, and Reproductive Sciences, School of Medicine, University of California, San Francisco, San Francisco, California, United States of America; 4 Department of Epidemiology, Mailman School of Public Health, Columbia University, New York, New York, United States of America; University of Sheffield, UNITED KINGDOM

## Abstract

**Background:**

Firearm dealer density is correlated with firearm interpersonal violence, but no quasi-experimental studies have assessed whether changes in dealer density lead to changes in firearm self-harm injuries and deaths. We assessed whether openings of firearm dealers are associated with short-term changes in local firearm self-harm injury rates.

**Methods:**

We identified 718 openings of firearm dealers in California using licensing data, 2014–2016. We defined exposure regions based on aggregations of zip codes defined by proximity to firearm dealer openings and matched each opening to four control regions on time and determinants of firearm injury. We applied a differences-in-differences approach to compare rates of firearm self-harm, in the month before and after each opening, in places with and without openings.

**Results:**

Firearm dealer openings were not associated with acute, local changes in firearm self-harm relative to places without openings (ratio of rate ratio: 0.90 [95% CI:0.68–1.19]). Results were robust to numerous sensitivity and secondary analyses.

**Conclusion:**

We found no associations of firearm dealer openings with acute, localized firearm self-harm deaths and injuries. Our focus on acute, local effects; broad availability of dealers and firearms; durability of firearms; or strong confounding-control may explain these null findings.

## Introduction

Firearms are a major means of self-harm and the most common method of suicide in the United States, accounting for approximately 25,000 suicides and 4,000 intentional self-harm injuries in in 2018 [1]. However, research on the causes of firearm self-harm and potential avenues for intervention remains limited [2]. Much research to date on prevention strategies has examined state-level policies [3, 4]. While this research has been valuable, local-level determinants of firearm self-harm are less often considered. Understanding local causes of firearm self-harm may help identify new prevention strategies [5].

Local retail environments are modifiable features of communities that shape health-related behaviors [6, 7]. For example, greater density of alcohol outlets contributes to alcohol consumption and alcohol-related harms [8, 9]. Firearm dealers are an important feature of the social ecology of firearm use, and proximity to firearm dealers may affect risk for firearm self-harm. Firearm dealers are the means by which a large portion of new firearms and ammunition are acquired [10]. Proximity to firearm dealers may imply greater access to firearms, ammunition, and related equipment, or greater exposure to firearm-related advertising, sales, discounts, or events. Each of these may promote firearm ownership among new or current gun owners or change firearm-related behavior—for example, by increasing the frequency with which firearms are used or how they are stored. Prior research supports links between firearm access and firearm ownership [4, 10], between firearm ownership and firearm suicide [11, 12], and between firearm-related events involving trainings, sales, and advertising and firearm injuries [13]. These relationships may apply to both legal and illegal transfers of firearms, as licensed dealers can be a source of straw purchases, in which an authorized purchaser buys on behalf of a prohibited person [14]. Suicides are particularly sensitive to firearm availability: greater firearm availability in the household or community, regardless of individual ownership, is associated with substantially increased risk of completed firearm suicide [4, 11, 15, 16]. Taken together, this evidence suggests that, across communities, within-community increases in firearm dealer density may lead to new firearm purchases and higher levels of firearm ownership, which in turn may imply greater availability of firearms for suicide completions in moments of crisis. Although suicide is a relatively rare event–especially when only narrow time intervals are considered–given their sensitivity to firearm availability, it is plausible that they may nonetheless be associated with increases in firearm dealer density at the population level.

To our knowledge, only one previous study has examined firearm dealers and self-harm. Chao and colleagues found a positive association of state-level firearm dealer density with firearm suicides [17], while Price et al found no association [18]. Local-level firearm dealer proximity or density, which are more likely to shape behavior, and nonfatal firearm self-harm injuries remain unexamined. Existing evidence on firearm homicides, robberies, gun-related crimes, or the proportion of suicides completed with firearms (a proxy for firearm ownership) suggests these outcomes are higher in areas with greater densities of firearm dealers [17, 19–23]. One concern with these cross-community comparisons is that confounding factors such as gun culture or business opportunity may determine both where firearm dealers are located and where firearm ownership or use may be higher. Although prior studies have controlled for community-level covariates such as demographic composition, many key confounders were not controlled. To our knowledge, no research on this topic has considered alternative study designs that may offer greater confounding control. No studies have examined acute changes in firearm dealer density, which can be operationalized as dealer openings. Given that overall firearm dealer density has remained unchanged over time in many places [17–23], leveraging such changes over time may strengthen causal inference.

We exploit variation in the timing and location of firearm dealer openings with a quasi-experimental, differences-in-differences design to investigate whether acute increases in firearm dealer density are associated with short-term increases in firearm suicides and self-harm injuries. This approach strengthens confounding control while focusing on effects immediately following and in close geographic proximity to openings.

## Materials and methods

### Overall approach

We used a quasi-experimental, difference-in-differences design [24, 25]. First, we compared rates of fatal and nonfatal firearm self-harm injuries (hereafter, combined as “firearm self-harm”) for the month immediately before and after firearm dealer openings, in communities proximal to each opening. Then, we compared this before-after difference for exposed communities to matched communities without dealer openings. This approach is advantageous, because we compare each dealer-community to itself over time, so potentially confounding community-level characteristics that are time-invariant over the comparison period are controlled by design, while differencing from comparable, time-matched communities without dealer openings controls for unmeasured time-varying factors that are shared between exposed and unexposed communities. Potentially confounding time-varying characteristics that are not shared are also less likely to change substantially over the short intervals under study. Further, our focus on acute changes in dealer availability also helps establish the time-ordering of firearm dealer increases and firearm self-harm.

### Firearm dealer data

In California, all firearm dealers are required to hold federal firearms licenses (FFLs). We compiled monthly data on California FFLs from the Bureau of Alcohol, Tobacco, Firearms, and Explosive’s (ATF) Federal Firearms Licensee Listings. Records are comprehensive, publicly available as of 2014, and include the geographic address of the licensee, license type, and month and year of license issuance. Multiple types of licenses exist; we restricted our study to FFLs that transfer firearms and ammunition, including licensees who transfer firearms but lack storefronts. We excluded licenses corresponding exclusively to “manufacturing”, “importing”, or “destructive devices other than firearms.” We assumed that an FFL “opened” in a month where a new license appeared for a physical address at which no licenses existed in the month prior. FFL data for September and October 2015 were not recorded due to a government shutdown; we treated these data as missing completely at random [26].

### Firearm injury data

We identified firearm self-harm from 2014–2016 using records of deaths, emergency department visits, and inpatient hospitalizations statewide from the California Department of Public Health Vital Records and the Office of Statewide Health Planning and Development. Records included the International Classification of Diseases (ICD), 9^th^ Revision Clinical Modification, 10^th^ Revision Clinical Modification, or 10^th^ Revision external cause of injury code and the residential zip code of the patient/decedent (see S1 Table for ICD codes in S1 File). External cause of injury coding in California’s hospital discharge records is required, subject to ongoing quality assurance, and regarded as 100% complete [27]. We did not examine firearm suicides alone or the proportion of suicides completed with a firearm because rates were too erratic over the time frames and geographic units studied to be examined separately.

### Matching

We identified control communities by matching the zip code of each opening to zip codes without a firearm dealer opening on key determinants of firearm self-harm at baseline [28]: month and year of the opening, hunting licenses per capita (California Department of Fish and Wildlife); population density, median income, percent veterans, median age, and percent non-Hispanic White (American Community Survey); density of other firearm dealers (firearm dealer data); and baseline rate of firearm violence (firearm injury data). We used the MatchIt package in R to apply greedy nearest neighbor propensity score matching without replacement to identify unique zip codes that were similar to the zip codes of openings with respect to the matched covariates [29]. For statistical efficiency, we matched four unexposed zip codes to each opening [30, 31]. We included a caliper of 0.1 standard deviations of the propensity score to enforce high-quality matches [32].

### Database construction

We constructed the analytic database at the level of the exposure region (aggregations of zip codes) and month (before or after the opening). Because FFLs are common, we hypothesized that openings would only influence small geographic areas in their immediate vicinity. Lacking evidence on the durations or distances over which FFLs might have effects, we defined a unique “exposure region” for each FFL opening (or control community) by combining the zip code of the FFL (or matched control zip code) with other zip codes with centroids within 5 kilometers (KM) of the FFL (see S1 Fig for illustration in S1 File). Within each exposure region, we calculated rates of firearm self-harm for the month before and after each FFL opening (or the FFL opening to which the control community was matched). Following previous research, we estimated rates per 100,000 individuals within these exposure regions using population denominators summed from Census-based zip code-month-specific estimates [5, 13].

Month units were the finest timescale available for FFL data. We hypothesized that some individuals may purchase firearms, ammunition, and related equipment and use them without much delay, and that we would primarily be able to detect acute changes that correspond to firearm use that is relatively immediate. Thus, our primary analysis compared the month before the opening to the month after; we excluded the month during because exposure classification during this period is mixed. We examine associations for this omitted period in a sensitivity analysis. Overall, these time frames and distances balance capturing short-term, localized effects with estimating stable rates. We tested the sensitivity of our results to the chosen timeframes and geographic areas in sensitivity analyses.

Although effects of FFL openings may persist for longer durations [33], we focus on acute effects to maximize internal validity. Specifically, time-varying, place-specific confounders such as economic prosperity are less likely to change substantially over the short periods under study. Further, our focus on acute changes in dealer availability helps establish the time-ordering of firearm dealer increases and firearm self-harm Short-term effects are also plausible: Evidence from gun shows suggests that acute effects of these events on firearm injuries can be detected within two weeks [13]. In addition, many suicidal behaviors are impulsive and short-lived, with completions strongly affected by firearm availability in moments of crisis [16]. Firearm purchases are associated with extremely elevated short-term risk of firearm suicide: as much as 57 times higher than the general population in the week following the purchase [12, 34]. Research on the association between alcohol outlet density and crime, which is similarly premised on how contextual availability increases purchases and use, has demonstrated that associations exist at multiple spatiotemporal scales but are most acutely identified at the level of the month and block group [35].

To avoid exposure misclassification due to the co-occurrence of multiple FFL openings in time and space, we excluded any cases of overlap. Specifically, overlap arises if two openings had overlapping geographic regions of exposure, and the “before” (unexposed) period of one opening (event B) overlapped with the “after” (exposed) period of an earlier opening (event A). In this situation, we excluded the overlapping zip codes from analyses of event B (hereafter, “overlap exclusions”). We also excluded rates that were extreme outliers (greater than 20 standard deviations from the mean).

### Covariates

We controlled for place-specific time-varying covariates that might be related to acute variations in firearm self-harm. These were monthly county-level unemployment rate (Bureau of Labor Statistics), precipitation, and average temperature (WestMap PRISM Climate Mapping Program).

### Differences-in-differences analysis

We used negative binomial regression to estimate associations of FFL openings with firearm self-harm using the lme4 package in R. Statistical testing of the dispersion parameter indicated that a negative binomial model fit the data well. We modeled the count of firearm self-harm injuries as a function of time (month before versus after the opening); an indicator of exposure to the opening (versus unexposed); an interaction between time and exposure status, an indicator for the transition from ICD-9-CM to ICD-10-CM on October 1, 2015; the time-varying place-specific covariates; random effects intercepts on exposure regions nested within cities; and an offset for the population of the exposure region. The primary association of interest was the exponentiated coefficient on the interaction term: the ratio of rate ratios (RRR) measuring the before-after change in firearm self-harm for places exposed to openings versus unexposed places. See S1 File for additional detail on the model specification.

Ethical review was conducted by the California Health and Human Services Agency and the University of California, Berkeley Committees for the Protection of Human Subjects.

### Sensitivity and secondary analyses

We conducted sensitivity tests varying the sizes of the time units and geographic areas of analysis. In addition, although the month during the opening includes a mixture of both time before and after the opening itself, we conducted sensitivity tests comparing firearm self-harm rates in the month during versus the month prior to openings, to evaluate whether any associations could be detected more immediately than in the month after openings.

In secondary analyses, we tested the hypothesis that the effects of FFL openings would be strongest for regions with no other nearby FFLs (in the same zip code) by restricting analyses to these regions. Additionally, because previous research identified variation in the associations of FFLs with firearm violence by urbanicity [19–23], we tested effect measure modification by urbanicity, classified as large cities, small cities/suburbs, and small towns/rural [36]. Further, we tested the association of dealer openings with nonfirearm self-harm, to test for potential substitution of means of self-harm from nonfirearm to firearm associated with potential increased firearm availability [37].

We measured our level of statistical power to detect effects, using an anticipated RRR of 1.15, assuming that FFL opening effects would be substantially smaller than those for gun shows (observed RRR 1.70 [13]). This analysis indicated that our study had 78% power to detect this change (see S1 File for details).

## Results

We identified 718 FFL openings during the study period. After excluding outlier rates and applying overlap exclusions, we included 580 openings with 2,272 controls in the main regression analyses (Table 1). The primary analytic dataset included 5,704 location-months based on 1.6 billion person-months of observation.

**Table 1:** Characteristics of firearm dealer openings and population exposure to these events.

Characteristic	N
Number of openings	718
Openings excluded due to overlap exclusions	115
Openings excluded due to extreme outlier rates	23
Final openings included in main regression analyses	580
Control places included in main regression analyses	2,272
Final openings and control places included in main regression analyses	2,852
Total person-months of observation in primary regression analyses	1,556,699,207

See “Database construction” for definition of overlap exclusions. Extreme outlier rates were defined as rates of size greater than 20 standard deviations from the mean.

Table 2 presents a summary of the characteristics of zip codes with and without FFL openings before and after matching (see S2 Table for further detail in S1 File). No openings were dropped due to inadequate matches but 36 of 2,812 controls were up-weighted to count as multiple controls when less than 4 adequate controls were identified. These weights were incorporated into all subsequent regression analyses.

**Table 2:** Comparison of median values of characteristics of communities exposed to firearm dealer openings versus matched comparison communities.

Characteristic	All California zip codes	Zip codes exposed to firearm dealer openings	Zip codes matched to those with firearm dealer openings
Number of zip codes	1,769	409	943
Population density (persons per square mile)	2,509	3,468	3,837
Veterans (%)	8	9	9
Median income ($)	57,202	60,853	60,832
Median age (years)	38	36	36
White, non-Hispanic (%)	58	59	55
Hunting licenses per 10,000 persons (N)	1,597	1,824	1,499
Baseline monthly rate of firearm injuries (per 100,000 persons)	0.8	1.0	0.9
Other firearm dealers in zip code (N)	0	4	3

This table summarizes the values of matching factors using medians. See S2 Table in S1 File for a full comparison of the distributions of characteristics of communities exposed to firearm dealer openings versus matched comparison communities.

Table 3 presents rates of firearm self-harm deaths and injuries, in months before and after FFL openings in at-risk regions local to these events, and the adjusted differences-in-differences estimates. Firearm self-harm decreased slightly following openings from 0.37 to 0.31 per 100,000 in regions exposed to these events. Adjusted estimates indicated that FFL openings were associated with a slight decrease in rates of firearm self-harm relative to places without openings (Table 2; RRR, 0.90 [95% CI:0.68–1.19]), but the confidence interval was wide.

**Table 3:** Crude and adjusted rates of firearm self-harm in the month before and after dealer openings in at-risk regions local to these events.

Time period and region	Measure	Value
Month before openings, places with openings	Total firearm self-harm injuries	128
	Rate per 100,000	0.37
Month after openings, places with openings	Total firearm self-harm injuries	117
	Rate per 100,000	0.31
Month after versus before openings, places without openings	Adjusted rate ratio (95% CI)	1.00 (0.88, 1.11)
Places with openings versus controls, month before openings	Adjusted rate ratio (95% CI)	0.86 (0.70, 1.05)
Adjusted ratio of rate ratios	Adjusted ratio of rate ratios (95% CI)	0.90 (0.68, 1.19)

Abbreviations: RRR: ratio of rate ratios. CI: confidence interval. Results in this table are based on the analytic data (at the level of the opening and month) after applying exclusions (see Methods). Adjusted RRR is based on a differences-in-differences analysis using negative binomial regression, adjusted for the October 1, 2015 transition from ICD-9-CM to ICD-10-CM; monthly county-level unemployment, temperature, and rainfall; and nested random effects intercepts on openings within cities.

Results of sensitivity analyses varying the sizes of the time units and geographic areas of analysis are presented in Table 4. Compared to the main analysis, associations were closer to the null and had similarly wide confidence intervals.

**Table 4:** Associations of firearm dealer openings with firearm self-harm for zip codes with no other firearm dealers, by urbanicity, for nonfirearm self-harm deaths and injuries, and for varied geographic ranges and durations of exposure.

Analytic specification	N	RRR (95% CI)
**20 KM geographic exposure range**	388 openings	0.97 (0.79,1.18)
**1 KM geographic exposure range**	678 openings	0.98 (0.69, 1.38)
**2-month exposure window**	494 openings	1.00 (0.74, 1.35)
**During vs. before openings**	580 openings	0.91 (0.69, 1.20)
**Zip codes with no other firearm dealers**	144 openings	0.81 (0.46, 1.43)
**Large cities**	470 openings	0.92 (0.69, 1.24)
**Small cities and suburbs**	81 openings	0.88 (0.34, 2.28)
**Small towns and rural**	29 openings	0.44 (0.04, 4.77)
**Nonfirearm self-harm injuries**	603 openings	1.00 (0.96, 1.04)

Abbreviations: N: number of openings included in analysis. RRR: ratio of rate ratios. CI: confidence interval.

In secondary analyses, restricting to openings without other nearby FFLs, no meaningful associations were observed (Table 4). Openings were not associated with changes in nonfirearm self-harm. When stratifying by rural-urban commuting area category (large cities, small cities and suburbs, and small towns/rural), results were suggestive of larger decreases in firearm self-harm in more rural areas and regions without other nearby FFLs, but these estimates were based on small numbers of events, so the confidence intervals were wide and crossed the null.

## Discussion

To our knowledge, this is the first study to assess whether openings of FFLs were associated with short-term increases in firearm self-harm in the communities where these openings occur. We found no meaningful overall associations of FFL openings with firearm self-harm. For rural areas and regions with no other nearby firearm dealers, results were suggestive of potential reductions in firearm self-harm associated with FFL openings, but estimates were too imprecise to draw conclusions.

Given our focus on local, short-term effects, we anticipated modest effect sizes. However, broad availability of firearms and FFLs may mean that changes in exposure to a single FFL have negligible impacts, especially because firearms are durable goods that can be used without replacement for long durations. Although FFL data do not include sales volumes, we sought to identify the highest-impact places by testing effects for localities without any other nearby FFLs. Results of this analysis were also null, which strengthens our confidence in the results.

Our hypothesis that FFL openings would lead to increases in firearm self-harm was based on prior studies that identified positive associations of firearm dealer density with firearm suicide, homicides, robberies, gun-related crimes, and firearm ownership [17–23]. However, there are several important differences between these studies and our approach. This was the first study to consider fatal and nonfatal firearm self-harm, which may be affected differently by FFLs. Previous research also examined long-term area-level FFL density; we assessed local, short-term changes in FFL density, operationalized as FFL openings. Prior studies employed regressions to control for measured confounders; we used a differences-in-differences design to control for unmeasured community-level confounders that are time-invariant over short comparison periods, unmeasured confounders that are time-varying but shared with matched comparison communities, and measured time-varying place-specific confounders. Our focus on acute changes in firearm self-harm may capture associations that are smaller in magnitude, but are more internally valid due to better confounding control. Our hypotheses were also based on evidence from gun shows, but these are larger events with potentially lower prices, less regulation, and greater influence than individual FFL openings [13].

Given that all studies involve tradeoffs and untestable assumptions [38], this study contributes to the literature on the potential influence of FFL density on rates of firearm injuries by employing a study design with distinct strengths and weaknesses compared to previous research on this topic. We tradeoff capturing the entire potential association of FFL density with firearm self-harm (which may unfold over long time periods and large geographic regions) with enhanced internal validity. We focus on the subset of the association, if one exists, that would plausibly be reflected over shorter time periods and smaller regions. Our approach better-limits the influence of potential confounders, and better-establishes temporal ordering, since FFLs may also open in places where rates of firearm availability and firearm injuries are already higher.

Our focus on California is important in interpreting the findings. California has some of the most restrictive firearm regulations in the country. For example, all firearm transfers in California are legally required to occur through an FFL, including transfers between private parties, and involve a 10-day waiting period. Although adherence and enforcement are undoubtedly imperfect, regulatory differences like this may mean that illegal or harmful activity leading to FFL-associated injuries may be less likely to occur in California. Supporting this, one prior study of Californians found that gun shows were associated with increases in firearm injuries for shows that occurred in Nevada, but not shows in California [13]. One proposed reason for this difference is the greater strength of California firearm laws. Given this, and that gun shows are comparatively much larger events than openings of individual FFLs, our finding of negligible associations of FFL openings with firearm self-harm is plausible. Future studies should test variation in associations of FFLs with firearm injuries by state firearm regulatory stringency.

Several additional limitations of this study must be noted. First, residual confounding is possible, although we sought to minimize bias through our study design. Although we controlled for unemployment rates, openings may reflect changes in the economic environment not captured by unemployment rates. Second, our identification of FFL openings relies on changes in dealer license status; delays or inaccuracies in reporting of license status or in correspondence between license issuance and dealer opening may have resulted in misclassification. Third, although we tested exposures over multiple geographic distances and time spans, it is possible that we have missed the most relevant exposure characterization. We estimated the overall association of FFL openings with firearm self-harm but dealer size, type (e.g. pawn shop, chain store), or sales volume may modify any associations. Fourth, we did not examine firearm homicide or assault outcomes because, given evidence that time and distance from firearm purchase to crime are often long [33], we hypothesized that these outcomes would only be affected by FFL density over longer durations and larger geographic ranges and thus were not amenable to our study design. Finally, our approach assumes that openings in one place did not affect outcomes in another place. This is a reasonable assumption, because we included overlap exclusions and openings in neighboring time and space were rare (e.g. 5% of openings occurred concurrently within 5KM).

In summary, we found no associations of firearm dealer openings with local, acute changes in firearm self-harm in California. This study adds to a small literature on firearm dealers as a feature of the local retail environment. Our findings differ from the five previous studies that have identified associations of firearm dealer density with firearm crime or injuries, possibly because we examined acute effects on firearm self-harm, or because our study design affords greater confounding control. Further research is warranted to determine the reasons for these differing findings, as well as the most relevant measures of firearm dealer availability.

## Supporting information

S1 File This file contains S1 Table, S2 Table, S1 Fig, and S2 Fig, as well as supplemental methods and results details in text. (DOCX)

## References

[1] Centers for Disease Control and Prevention. CDC Web-based Injury Statistics Query and Reporting System (WISQARS). 3 Dec 2018 [cited 5 Dec 2018]. Available: https://www.cdc.gov/injury/wisqars/index.html

[2] Dzau VJ, Leshner AI. Public Health Research on Gun Violence: Long Overdue. Annals of Internal Medicine. 2018;168: 876. 10.7326/M18-0579 29554693

[3] Galea S, Branas CC, Flescher A, Formica MK, Hennig N, Liller KD, et al. Priorities in Recovering From a Lost Generation of Firearms Research. Am J Public Health. 2018;108: 858–860. 10.2105/AJPH.2018.304436 29874487 PMC5993412

[4] RAND Corporation. The Science of Gun Policy: A Critical Synthesis of Research Evidence on the Effects of Gun Policies in the United States. Santa Monica, CA; 2018. Available: https://www.rand.org/pubs/research_reports/RR2088.html 10.7249/rhq-08-01-05 PMC6075800 30083426

[5] Matthay EC, Farkas K, Skeem J, Ahern J. Exposure to Community Violence and Self-harm in California: A Multilevel, Population-based, Case–Control Study. Epidemiology. 2018;29: 697. 10.1097/EDE.0000000000000872 29889134 PMC6066408

[6] Ashe M, Jernigan D, Kline R, Galaz R. Land Use Planning and the Control of Alcohol, Tobacco, Firearms, and Fast Food Restaurants. American Journal of Public Health. 2003;93: 1404–1408. 10.2105/ajph.93.9.1404 12948952 PMC1447982

[7] Peterson RD, Krivo LJ, Harris MA. Disadvantage and Neighborhood Violent Crime: Do Local Institutions Matter? Journal of Research in Crime and Delinquency. 2000;37: 31–63. 10.1177/0022427800037001002

[8] Gruenewald PJ. Regulating Availability: How Access to Alcohol Affects Drinking and Problems in Youth and Adults. Alcohol Res Health. 2011;34: 248–256. 22330225 PMC3860569

[9] Campbell CA, Hahn RA, Elder R, Brewer R, Chattopadhyay S, Fielding J, et al. The effectiveness of limiting alcohol outlet density as a means of reducing excessive alcohol consumption and alcohol-related harms. Am J Prev Med. 2009;37: 556–569. 10.1016/j.amepre.2009.09.028 19944925

[10] Miller M, Hepburn L, Azrael D. Firearm Acquisition Without Background Checks: Results of a National Survey. Ann Intern Med. 2017;166: 233. 10.7326/M16-1590 28055050

[11] Anglemyer A, Horvath T, Rutherford G. The Accessibility of Firearms and Risk for Suicide and Homicide Victimization Among Household Members: A Systematic Review and Meta-analysis. Annals of Internal Medicine. 2014;160: 101–110. 10.7326/M13-1301 24592495

[12] Studdert DM, Zhang Y, Swanson SA, Prince L, Rodden JA, Holsinger EE, et al. Handgun Ownership and Suicide in California. New England Journal of Medicine. 2020;382: 2220–2229. 10.1056/NEJMsa1916744 32492303

[13] Matthay EC, Galin J, Rudolph KE, Farkas K, Wintemute GJ, Ahern J. In-State and Interstate Associations Between Gun Shows and Firearm Deaths and Injuries: A Quasi-experimental Study. Annals of Internal Medicine. 2017;167: 837. 10.7326/M17-1792 29059689 PMC5972533

[14] Sorenson SB, Vittes KA. Buying a handgun for someone else: firearm dealer willingness to sell. Injury Prevention. 2003;9: 147–150. 10.1136/ip.9.2.147 12810742 PMC1730960

[15] Westefeld J, Gann L, Lustgarten S, Yeates K. Relationships between firearm availability and suicide: The role of psychology. Professional Psychology: Research and Practice. 2016;47: 271–277.

[16] Miller M, Azrael D, Barber C. Suicide mortality in the United States: The importance of attending to method in understanding population-level disparities in the burden of suicide. 2012. 10.1146/annurev-publhealth-031811-124636 22224886

[17] Chao SD, Kastenberg ZJ, Madhavan S, Staudenmayer K. Impact of licensed federal firearm suppliers on firearm-related mortality. Journal of Trauma and Acute Care Surgery. 2019;86: 123–127. 10.1097/TA.0000000000002067 30212424

[18] Price JH, Thompson AJ, Dake JA. Factors Associated with State Variations in Homicide, Suicide, and Unintentional Firearm Deaths. Journal of Community Health. 2004;29: 271–283. 10.1023/b:johe.0000025326.89365.5c 15186014

[19] Steidley T, Ramey DM, Shrider EA. Gun Shops as Local Institutions: Federal Firearms Licensees, Social Disorganization, and Neighborhood Violent Crime. Soc Forces. 2017;96: 265–298. 10.1093/sf/sox039

[20] Wiebe DJ, Krafty RT, Koper CS, Nance ML, Elliott MR, Branas CC. Homicide and geographic access to gun dealers in the United States. BMC Public Health. 2009;9: 199. 10.1186/1471-2458-9-199 19549293 PMC2714509

[21] Stansfield R, Semenza D. Licensed firearm dealer availability and intimate partner homicide: A multilevel analysis in sixteen states. Prev Med. 2019;126: 105739. 10.1016/j.ypmed.2019.05.027 31152829

[22] Xu Y, Owusu-Agyemang S. Gun-Related Crime in Detroit, Michigan: Exploring the Spatial Context of Licensed Firearm Availability and Neighborhood Characteristics. Papers in Applied Geography. 2019;0: 1–19. 10.1080/23754931.2019.1619192

[23] Semenza DC, Stansfield R, Link NW. The Dynamics of Race, Place, and Homicide Context in the Relationship between Firearm Dealers and Gun Violence. Justice Quarterly. 2020;0: 1–18. 10.1080/07418825.2019.1707858

[24] Angrist JD, Pischke J-S. Mostly harmless econometrics: An empiricist’s companion. Princeton University Press; 2008.

[25] Dimick JB, Ryan AM. Methods for Evaluating Changes in Health Care Policy: The Difference-in-Differences Approach. JAMA. 2014;312: 2401–2402. 10.1001/jama.2014.16153 25490331

[26] Ibrahim JG, Chu H, Chen M-H. Missing Data in Clinical Studies: Issues and Methods. J Clin Oncol. 2012;30: 3297–3303. 10.1200/JCO.2011.38.7589 22649133 PMC3948388

[27] Centers for Disease Control and Prevention. Strategies to Improve External Cause-of-Injury Coding in State-Based Hospital Discharge and Emergency Department Data Systems: Recommendations of the CDC Workgroup for Improvement of External Cause-of-Injury Coding. Morbidity and Mortality Weekly Report. 2008;57: 1–16. 18368008

[28] Hawton K, van Heeringen K. Suicide. The Lancet. 2009;373: 1372–1381. 10.1016/S0140-6736(09)60372-X 19376453

[29] Ho D, Imai K, King G, Stuart E. MatchIt: Nonparametric Preprocessing for Parametric Causal Inference. Journal of Statistical Software. 2011;42.

[30] Wacholder S, Silverman DT, McLaughlin JK, Mandel JS. Selection of Controls in Case-Control Studies: III. Design Options. Am J Epidemiol. 1992;135: 1042–1050. 10.1093/oxfordjournals.aje.a116398 1595690

[31] Rose S, van der Laan MJ. Why Match? Matched Case-Control Studies. In: van der Laan MJ, Rose S, editors. Targeted Learning: Causal Inference for Observational and Experimental Data. New York, NY: Springer; 2011. pp. 229–238. 10.1007/978-1-4419-9782-1_14

[32] Austin PC. Optimal caliper widths for propensity-score matching when estimating differences in means and differences in proportions in observational studies. Pharmaceutical Statistics. 2011;10: 150–161. 10.1002/pst.433 20925139 PMC3120982

[33] Wintemute GJ, Romero MP, Wright MA, Grassel KM. The life cycle of crime guns: A description based on guns recovered from young people in california. Annals of Emergency Medicine. 2004;43: 733–742. 10.1016/S0196064403012241 15159705

[34] Wintemute GJ, Parham CA, Beaumont JJ, Wright M, Drake C. Mortality among recent purchasers of handguns. New England Journal of Medicine. 1999;341: 1583–1589. 10.1056/NEJM199911183412106 10564689

[35] Mair C, Sumetsky N, Gaidus A, Gruenewald PJ, Ponicki WR. Multiresolution Analyses of Neighborhood Correlates of Crime: Smaller Is Not Better. American Journal of Epidemiology. 2021;190: 150–160. 10.1093/aje/kwaa157 32700726 PMC7784528

[36] USDA ERS—Rural-Urban Commuting Area Codes. [cited 7 Feb 2020]. Available: https://www.ers.usda.gov/data-products/rural-urban-commuting-area-codes/

[37] Daigle MS. Suicide prevention through means restriction: Assessing the risk of substitution: A critical review and synthesis. Accident Analysis & Prevention. 2005;37: 625–632. 10.1016/j.aap.2005.03.004 15949453

[38] Matthay EC, Hagan E, Gottlieb LM, Tan M, Vlahov D, Adler NE, et al. Alternative Causal Inference Methods in Population Health Research: Evaluating Tradeoffs and Triangulating Evidence. SSM—Population Health. 2019;10: 100526. 10.1016/j.ssmph.2019.100526 31890846 PMC6926350

