# Supplementary material for: Associations of firearm dealer openings with firearm self-harm deaths and injuries: A differences-in-differences analysis
Source: PLoS One. 2021 Mar 18;16(3):e0248130. doi: 10.1371/journal.pone.0248130 (PMC7971548; doi:10.1371/journal.pone.0248130)
Supplement: S1 File — (DOCX) [file pone.0248130.s001.docx]

**Supporting Information**

**Supplemental methods**

**S1 Table. International Classification of Diseases (ICD) 9^th^ and 10^th^ revision external cause of injury codes used to classify firearm and nonfirearm self-harm deaths and injuries.**

| **Means of death or injury** | **ICD-9-CM***  (hospital discharge records, January 2014 – September 2015) | **ICD-10-CM***  (hospital discharge codes, October 2015 – December 2016) | **ICD-10***  (mortality records, all time periods) |
| --- | --- | --- | --- |
| Intentional firearm self-harm | E9550 – E9554 | X72, X73, X748, X749 | X72, X73 |
| Intentional nonfirearm self-harm | E950 – E954, E9555 – E9557, E9559, E956 – E958 | X71, X740, X75 – X83, T36 – 65**, T71**, T1491 | X60 – X71, X74 – X84, U3 |

* Late effects and sequela of firearm and nonfirearm intentional self-harm were excluded to avoid double counting of incidents and for consistent classification across the ICD-9-CM to ICD-10-CM transition.

** For nonfirearm intentional self-harm, ICD-10-CM codes T36-T65 and T71 for poisonings and asphyxiation (all subtypes) were those with a “2” specified for the fifth or sixth digit of each code (indicating intentional self-harm).

**Exposure definition**

S1 Fig illustrates our approach to defining exposure regions specific to each firearm dealer opening. The map displays a region in central California and the black lines indicate the boundaries of the zip codes. The red “X” indicates the location of a hypothetical firearm dealer opening. The black crosses indicate the geographic centroids of each zip code. The blue cross indicates the geographic centroid of the zip code in which the firearm dealer opening occurred. The blue circle indicates the 5-kilometer radius around the geographic centroid of the zip code in which the firearm dealer opening occurred. We defined the exposure region as the zip code in which the opening occurred, plus all zip codes with centroids within 5 kilometers of the centroid of the opening zip code. Thus, the exposure region is made up of all the zip codes with centroids (black crosses) inside the blue circle. For the hypothetical firearm dealer opening marked by the red “X”, the corresponding exposure region is outlined in yellow.

S2 Fig displays the locations of all identified firearm dealer openings across California, 2014-2016. Some occurred close to one another in space but at different times.

**S1 Fig. Illustration of approach to defining exposure regions specific to each firearm dealer opening.**

**S2 Fig. Locations of firearm dealer openings, California, 2014-2016.**

**Differences-in-differences model specification**

We used multivariable mixed effects regressions to estimate associations of FFL openings with firearm self-harm. Statistical testing of the dispersion parameter indicated that a negative binomial model fit the data well. We used the specification:

$$log\left( Y_{tec} \right)=\beta_{0}+ \beta_{1}T+{\beta_{2}D+\beta_{3}TD+ \beta}_{4}I+\beta_{5}X_{tec}+\rho_{ce} +\rho_{c}+\log\left( d_{tec} \right)+\varepsilon_{tec}$$

where $Y_{tec}$ was the count of deaths and injuries at time $t$, in the region surrounding opening $e$ located in city $c$; $\beta_{0}$, the intercept; $T$, an indicator for the month after the opening (versus before); $D$, an indicator of exposure to the opening (versus control) ; $I$, an indicator to account for the transition from ICD-9-CM to ICD-10-CM on October 1, 2015; $X_{tec}$, the matrix of time-varying place-specific covariates; $\rho_{ce}$ and $\rho_{c}$, random effects intercepts to account for clustering by opening exposure nested within cities; $\log\left( d_{tec} \right)$, an offset for the population of the exposure region; and $\varepsilon_{tec}$, the error term. The primary association of interest was the ratio of rate ratios (RRR) measuring the before-after change in firearm self-harm for places exposed to openings versus unexposed places ($\exp\left( \beta_{3} \right)$). Regression analysis was conducted using the lme4 package in R.

**Statistical power**

We measured our level of statistical power to detect effects using the power.nb.test function of the MKmisc package in R for two negative binomial rates. We input baseline outcome rates from the observed data, negative binomial theta parameters from the primary analysis initialization, fixed samples sizes of openings and matched control communities measured at two time points, a one-sided alternative hypothesis, and statistical significance level of 0.05. We used an anticipated RRR of 1.15, assuming that FFL opening effects would be substantially smaller than those for gun shows (observed RRR 1.70 [13]). This analysis indicated that our study had 78% power to detect this change.

**Supplemental results**

**S2 Table. Distribution of characteristics of communities exposed to firearm dealer openings and matched comparison communities.**

| **Characteristic** | **Metric** | **All California zip codes** | **Zip codes exposed to firearm dealer openings** | **Zip codes matched to those with firearm dealer openings** |
| --- | --- | --- | --- | --- |
| Zip codes | Number | 1769 | 409 | 943 |
| Population density (persons per square mile) | Minimum | 1 | 1 | 1 |
|  | 25^th^ percentile | 43 | 1,549 | 1,592 |
|  | Median | 2,509 | 3,468 | 3,837 |
|  | 75^th^ percentile | 6,906 | 5,754 | 5,993 |
|  | Maximum | 113,893 | 26,135 | 31,065 |
| Veterans (%) | Minimum | 0 | 1 | 0 |
|  | 25^th^ percentile | 5 | 7 | 6 |
|  | Median | 8 | 9 | 9 |
|  | 75^th^ percentile | 12 | 12 | 12 |
|  | Maximum | 100 | 29 | 44 |
| Median income ($) | Minimum | 9219 | 14,452 | 11,731 |
|  | 25^th^ percentile | 42,544 | 47,366 | 46,916 |
|  | Median | 57,202 | 60,853 | 60,832 |
|  | 75^th^ percentile | 76,727 | 81,660 | 78,923 |
|  | Maximum | 240,833 | 240,833 | 240,883 |
| Median age (years) | Minimum | 8 | 20 | 16 |
|  | 25^th^ percentile | 32 | 32 | 32 |
|  | Median | 38 | 36 | 36 |
|  | 75^th^ percentile | 45 | 40 | 40 |
|  | Maximum | 88 | 61 | 74 |
| White, non-Hispanic (%) | Minimum | 0 | 2 | 0 |
|  | 25^th^ percentile | 32 | 39 | 36 |
|  | Median | 58 | 59 | 55 |
|  | 75^th^ percentile | 78 | 72 | 69 |
|  | Maximum | 100 | 100 | 100 |
| Hunting licenses per 10,000 persons (N) | Minimum | 0 | 55 | 41 |
|  | 25^th^ percentile | 508 | 810 | 741 |
|  | Median | 1,597 | 1,824 | 1,499 |
|  | 75^th^ percentile | 5,296 | 4,454 | 4,132 |
|  | Maximum | 1,340,929 | 29,810 | 41,221 |
| Baseline rate of firearm injuries (per 100,000 persons) | Minimum | 0.0 | 0.0 | 0.0 |
|  | 25^th^ percentile | 0.2 | 0.6 | 0.6 |
|  | Median | 0.8 | 1.0 | 0.9 |
|  | 75^th^ percentile | 1.9 | 1.7 | 1.6 |
|  | Maximum | 1349.1 | 9.7 | 19.7 |
| Other firearm dealers in zip code (N) | Minimum | 0 | 0 | 0 |
|  | 25^th^ percentile | 0 | 2 | 2 |
|  | Median | 0 | 4 | 3 |
|  | 75^th^ percentile | 2 | 6 | 6 |
|  | Maximum | 24 | 10 | 10 |
